# The Cellular Distribution of RanGAP1 Is Regulated by CRM1-Mediated Nuclear Export in Mammalian Cells

**DOI:** 10.1371/journal.pone.0141309

**Published:** 2015-10-27

**Authors:** Keith Cha, Progga Sen, Sarita Raghunayakula, Xiang-Dong Zhang

**Affiliations:** Department of Biological Sciences, Wayne State University, Detroit, Michigan, United States of America; University of Toronto, CANADA

## Abstract

The Ran GTPase activating protein RanGAP1 plays an essential role in nuclear transport by stimulating RanGTP hydrolysis in the cytoplasmic compartment. In mammalian cells, unmodified RanGAP1 is predominantly cytoplasmic, whereas modification by small ubiquitin-related modifier protein (SUMO) targets RanGAP1 to the cytoplasmic filaments of nuclear pore complex (NPC). Although RanGAP1 contains nine putative nuclear export signals and a nuclear localization signal, little is known if RanGAP1 shuttles between the nuclear and cytoplasmic compartments and how its primary localization in the cytoplasm and at the NPC is regulated. Here we show that inhibition of CRM1-mediated nuclear export using RNAi-knockdown of CRM1 and inactivation of CRM1 by leptomycin B (LMB) results in nuclear accumulation of RanGAP1. LMB treatment induced a more robust redistribution of RanGAP1 from the cytoplasm to the nucleoplasm compared to CRM1 RNAi and also uniquely triggered a decrease or loss of RanGAP1 localization at the NPC, suggesting that LMB treatment is more effective in inhibiting CRM1-mediated nuclear export of RanGAP1. Our time-course analysis of LMB treatment reveals that the NPC-associated RanGAP1 is much more slowly redistributed to the nucleoplasm than the cytoplasmic RanGAP1. Furthermore, LMB-induced nuclear accumulation of RanGAP1 is positively correlated with an increase in levels of SUMO-modified RanGAP1, suggesting that SUMOylation of RanGAP1 may mainly take place in the nucleoplasm. Lastly, we demonstrate that the nuclear localization signal at the C-terminus of RanGAP1 is required for its nuclear accumulation in cells treated with LMB. Taken together, our results elucidate that RanGAP1 is actively transported between the nuclear and cytoplasmic compartments, and that the cytoplasmic and NPC localization of RanGAP1 is dependent on CRM1-mediated nuclear export.

## Introduction

The Ras-like GTPase Ran plays an essential role in various cellular processes including nuclear transport, mitotic spindle assembly, and nuclear envelope reformation [1–5]. Like many other small GTPases, Ran cycles between its GTP- and GDP-bound states and thus functions as a molecular switch. However, Ran is unable to exchange between the two states at a physiologically significant rate by itself and requires interaction with two essential regulators, the Ran GTPase-activating protein RanGAP and the Ran guanine nucleotide exchange factor RanGEF (also called RCC1) [6–8]. RanGAP accelerates the hydrolysis of RanGTP to RanGDP by ~10^5^ fold, and RanGEF increases the GDP/GTP exchange on Ran by the same factor [9]. Because RanGAP is primarily cytoplasmic whereas RCC1 is exclusively nuclear, this asymmetry creates a steep concentration gradient from high RanGTP levels in the nucleoplasm to low RanGTP levels in the cytoplasm [10]. This gradient provides the driving force for nuclear transport of numerous proteins and RNAs across the nuclear pore complex (NPC) at the nuclear envelope [2].

This Ran-driven nuclear transport is mediated by a family of nuclear transport receptors known as karyopherins which includes both importins and exportins [1, 2]. Importin binds to the nuclear localization signal (NLS) of a cargo in the cytoplasm and then releases it upon the interaction with RanGTP in the nucleoplasm [1, 2]. The Importin-RanGTP complex exits from the nucleoplasm and then dissociates upon RanGTP hydrolysis activated by RanGAP along with its accessory factor RanBP1 or RanBP2 (also known as Nup358) in the cytoplasm. The sum of these events leads to the recycling of Importin for the next round of nuclear import. Conversely, Exportin binds to the nuclear export signal (NES) of a cargo in the presence of RanGTP in the nucleoplasm and subsequently releases the cargo upon RanGTP hydrolysis mediated by RanGAP and RanBP1 or RanBP2 in the cytoplasm. Hence, the predominantly cytoplasmic localization of RanGAP is not only required for establishing the RanGTP gradient but also for disassembling the Importin-RanGTP and cargo-Exportin-RanGTP complexes in the right subcellular compartment.

The RanGAP proteins from various organisms are characterized by an N-terminal leucine-rich repeat domain (LRR) (~330–350 residues) followed by an acidic region (~40 residues) [11]. Compared to the yeast RanGAP (known as Rna1p) from *S*. *pombe* and *S*. *cerevisiae*, the vertebrate RanGAP1 from human, mouse and *Xenopus* contains an additional C-terminal domain (~230 residues) [11–13]. Moreover, vertebrate RanGAP1 is covalently modified by SUMO1 at a conserved lysine (K) residue within its C-terminal SUMO-attachment domain (SUMO-AD) [14, 15]. While unmodified RanGAP1 is primarily cytoplasmic, SUMO-modification of RanGAP1 targets it to the cytoplasmic filaments of the NPC by forming a stable complex with RanBP2 and Ubc9 [16–19]. Among the three vertebrate SUMO paralogs, SUMO2 and SUMO3 (referred to as SUMO-2/3) are ~96% identical to each other, but they share only ~45% identity to SUMO1. In spite of being equally modified by SUMO1 and SUMO2 *in vitro*, RanGAP1 is preferentially modified by SUMO1 *in vivo* [14, 15]. SUMO1-modified RanGAP1 forms a more stable complex with RanBP2 and Ubc9 and therefore better protected from isopeptidase-mediated deSUMOylation when compared to SUMO2-modified RanGAP1 [18].

Mammalian RanGAP1 contains a non-classical NLS at its C-terminal SUMO-AD domain and nine putative leucine-rich NESs, which can be recognized by CRM1 (also known as Exportin 1 or Xpo1), at its N-terminal LRR domain [12]. CRM1 is a major conserved exportin and mediates the export of proteins containing a leucine-rich NES [20–26]. This raises a possibility that mammalian RanGAP1 may be actively transported into and out of the nucleoplasm by Importin and Exportin (such as CRM1). However, little is known if mammalian RanGAP1 shuttles between the nuclear and cytoplasmic compartments and how its primary localization in the cytoplasm and at the NPC is regulated. In this study, we show that inhibition of CRM1-mediated nuclear export causes a nuclear accumulation of RanGAP1 along with a decrease or loss of RanGAP1 in the cytoplasm and at the NPC in mammalian cells. In addition, the nuclear accumulation of RanGAP1 is associated with an increase in SUMOylation of RanGAP1. Lastly, we demonstrate that the NLS sequence at the C-terminus of RanGAP1 is required for its nuclear import.

## Materials and Methods

### Antibodies

Antibodies used in this study include: mouse anti-RanGAP1 (19C7) monoclonal antibody (mAb) [14] from Dr. Michael Matunis (Johns Hopkins University, Baltimore, MD); mouse anti-Myc (9E10) mAb (sc-40; Santa Cruz); rabbit anti-Myc polyclonal antibody (2272; Cell Signaling); rabbit anti-RanBP2 polyclonal antibody (ab64276; Abcam); mouse mAb414 (MMS-120R; Covance); mouse anti-CRM1 mAb (611832; BD Biosciences).

### Plasmids and siRNAs

The pcDNA3-Myc-RanGAP1 plasmid encoding Myc-tagged mouse RanGAP1 wild-type (WT) (1–589) was provided by Dr. Michael Matunis [12]. The plasmids encoding the C-terminal deletion mutants of Myc-RanGAP1, CΔ23 (1–566) and CΔ49 (1–540), were constructed using the following strategy. A forward PCR primer (ATTGGTACCGAGCTCGGATCCACTAG) complementary to the pcDNA3 multiple cloning site and with a KpnI site was paired with one of the reverse PCR primers (GCTCTAGACTATGTCACAAATGCCAA and GCTCTAGACTAGG GGCCATGCAGGCT) complementary to the appropriate RanGAP1 coding sequences and a XbaI site for amplifying the sequences encoding Myc-tagged RanGAP1-CΔ23 and RanGAP1-CΔ49, respectively. The amplified PCR fragments were cloned into the KpnI and XbaI sites of the pcDNA3-Myc-RanGAP1 plasmid to replace the insert coding Myc-RanGAP1 WT. All the constructs were verified by DNA sequencing. Two CRM1-specific siRNA oligonucleotides, siRNA 1 (5’-UGUGGUGAAUUGCUUAUAC-3’) [27, 28] and siRNA 2 (5’-GGAACCAGU GCGAAGGAAU-3’), the siRNAs specific to RanBP2 (CACAGAC AAAGCCG UUGAA) [29], and the non-targeting control siRNA oligonucleotides (5’-UUCUCCGAA CGUGUCACGU-3’) [30] were purchased from Dharmacon.

### Cell culture, treatment and transfection

Human cervical cancer cells (HeLa) [31] and Buffalo rat liver cells (BRL) [14] were cultured in Dulbecco's Modified Eagle's Medium (DMEM) (Hyclone) with 10% fetal bovine serum (FBS) (Hyclone) and 1% penicillin-streptomycin antibiotics (Invitrogen). A 1000× stock solution of 20 μM leptomycin B (LMB) (Sigma-Aldrich) was prepared in Phosphate-buffered saline (PBS) containing 0.3% DMSO. To inhibit CRM1-mediated nuclear export, the LMB stock solution was added to culture media for treating cells with a final concentration of 20 nM LMB [32, 33] for the indicated times, whereas PBS containing 0.3% DMSO was used as a control.

Three different methods were used for transfection. First, the calcium phosphate method [34] was used to transfect cells with the plasmids encoding Myc-RanGAP1 WT and mutant for immunofluorescence microscopy. Second, Lipofectamine-Plus reagent (Invitrogen) was used for transfecting cells with the plasmids encoding Myc-RanGAP1 WT and mutant for immunoblot analysis. Third, Oligofectamine (Invitrogen) was used for transfecting cells with control siRNA or siRNA against CRM1 or RanBP2. Notably, levels of Myc-RanGAP1 in cells transfected using the calcium phosphate method were considerably lower than those in cells transfected using Lipofectamine-Plus reagent (Invitrogen) and also closer to levels of endogenous RanGAP1 (data not shown). To accurately measure the subcellular distribution of Myc-RanGAP1, we therefore used the calcium phosphate method for transfecting cells with the plasmids encoding Myc-RanGAP1 for immunofluorescence analysis.

To evaluate the efficiency of RNA interference (RNAi) for inhibiting CRM1 expression, HeLa cells were transfected with control or CRM1-specific siRNAs for 48 h and then analyzed by immunoblotting with antibodies specific to CRM1 and α-tubulin. To examine if CRM1 RNAi affects the localization of Myc-RanGAP1, HeLa cells were transfected with control or CRM1-specific siRNAs for 24 h and then with the plasmid encoding Myc-tagged RanGAP1 for another 24 h followed by immunofluorescence analysis using anti-Myc mAb (9E10). To test if the SUMO E3 ligase RanBP2 is required for the increase of RanGAP1 SUMOylation in cells with LMB treatment, HeLa cells were transfected with control siRNA or siRNA specific to RanBP2 for 72 h and treated with LMB or a control solution for 8 h followed by immunoblot analysis with antibodies specific to RanGAP1, RanBP2 and α-tubulin.

### Immunofluorescence microcopy and image analysis

BRL and HeLa cells were fixed with 3.5% paraformaldehyde in PBS for 30 min, permeabilized with 0.5% Triton X-100 in PBS for 5 min, and then stained with primary antibodies for 1 h and Alexa Fluor 488- and/or 594-conjugated secondary antibodies (Invitrogen) for 30 min followed by incubation with a mounting solution containing 4′,6-diamidino-2 phenylindole (DAPI) for 5 min. Immunofluorescence images were taken using the inverted Olympus IX81 fluorescence microscope with U-Plan S-Apo 60×/1.35 NA oil immersion objective and acquired with the MicroSuite acquisition software (Olympus). The immunofluorescence signal intensities of Myc-tagged RanGAP1 in the nucleoplasm and the cytoplasm were measured by ImageJ software (NIH). To calculate the mean nuclear to cytoplasmic signal ratio (N/C) of Myc-RanGAP1 and its presence or absence at the NPC, 50 cells for each siRNA and for each time point of LMB treatment/removal from each of the three independent experiments were analyzed. The N/C ratios were classified into <1, 1–2, or >2. Each bar represents the mean value ± SEM (Student’s *t* test).

## Results

### RNAi-mediated knockdown of CRM1 causes nuclear accumulation of RanGAP1 in mammalian cells

**Fig 1:**
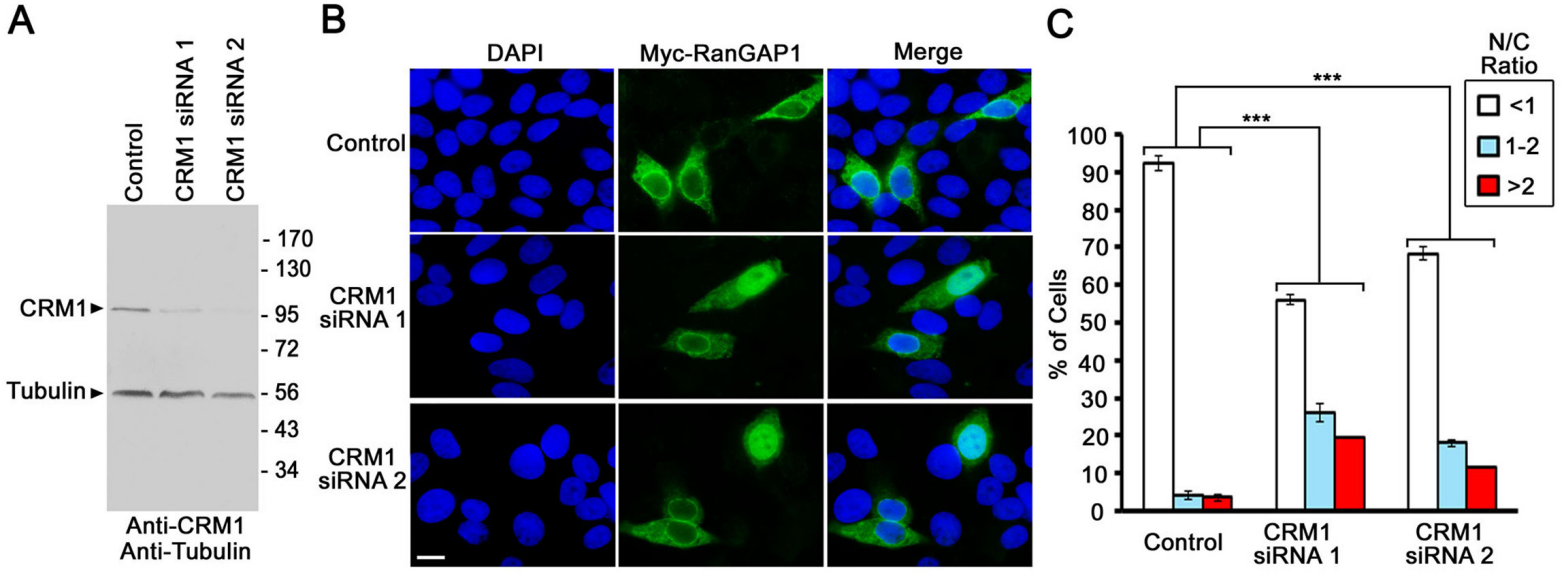
RNAi-mediated depletion of CRM1 results in nuclear accumulation of RanGAP1 in mammalian cells. (A) HeLa cells were transfected with control siRNA or CRM1-specific siRNA 1 or 2 for 48 h followed by immunoblotting using antibodies specific CRM1 and α-tubulin. (B) HeLa cells were transfected with control siRNA or one of the two CRM1-specific siRNAs for 24 h and then the plasmids encoding Myc-tagged mouse RanGAP1 for 24 h followed by immunofluorescence microscopy analysis with mouse anti-Myc mAb (9E10). Bar, 10 μm. (C) The histogram shows the percentage of cells exhibiting the mean nuclear to cytoplasmic concentration ratio (N/C) of Myc-RanGAP1 in control or CRM1 RNAi cells. The immunofluorescence signal intensities of Myc-RanGAP1 in the nucleoplasm and cytoplasm were measured by ImageJ software (NIH) using cells from three independent experiments. Each bar represents the mean percentage value of cells with the indicated N/C ratio ± SEM for each treatment (*N* = 40, ***P < 0.001, Student’s *t* test).

Here we hypothesized that CRM1-mediated nuclear export is required for the primarily cytoplasmic distribution of RanGAP1 in mammalian cells. To test this, we first established an RNAi approach to efficiently knock down CRM1 expression. To this end, HeLa cells were transfected with control siRNA or CRM1-specific siRNA 1 or 2 and then analyzed by immunoblotting with anti-CRM1 and anti-tubulin antibodies. Compared to control siRNA, both CRM1-specific siRNA 1 or 2 resulted in an over 70% reduction of CRM1 protein expression (Fig 1A). To examine the effect of CRM1 RNAi on the subcellular distribution of RanGAP1, HeLa cells were transfected with control or CRM1-specific siRNAs for 24 h and then the plasmid encoding Myc-tagged RanGAP1 [12] for another 24 h followed by immunofluorescence microscopy using anti-Myc mAb (9E10). Consistent with previous studies [13], we observed a high cell to cell variation in levels of Myc-RanGAP1 in the transfected cells. To more accurately determine the subcellular distribution of Myc-RanGAP1, we analyzed cells with an intermediate level of staining intensity of Myc-RanGAP1 by excluding the cells with the highest or lowest ~10–15% expression of Myc-RanGAP1.

Compared to control RNAi, CRM1 RNAi using either siRNA 1 or 2 significantly increased the percentage of cells with a nuclear accumulation of Myc-RanGAP1 as indicated by a high nuclear to cytoplasmic concentration ratio (N/C) of Myc-RanGAP1 of ≥1 (including 1–2 and >2) (*N* = 40, ***P < 0.001) (Fig 1B and 1C). While only ~7% (1–2: 4%; >2: 3%) of control RNAi cells displayed an N/C ratio of ≥1, ~45% (1–2: 26%; >2: 19%) and ~30% (1–2: 18%; >2: 12%) of CRM1 RNAi cells respectively transfected with siRNA 1 and 2 exhibited an N/C ratio of ≥1 (Fig 1C). However, we also noted that >50% of CRM1 RNAi cells still showed a largely cytoplasmic distribution of Myc-RanGAP1 with an N/C ratio of <1 (Fig 1B and 1C). This might be caused by incomplete knockdown of CRM1 in these cells as indicated by our immunoblot results (Fig 1A). Hence, taken together our results supported the hypothesis that the cytoplasmic localization of RanGAP1 is dependent on CRM1-mediated nuclear export in mammalian cells.

### Inhibition of CRM1-mediated export by LMB causes a redistribution of RanGAP1 from the cytoplasm and the NPC to the nucleoplasm

To further test if CRM1 is required for the cytoplasmic distribution of RanGAP1, we used another well-established approach to inhibit CRM1-mediated export by treating cells with a highly potent and specific inhibitor of CRM1 called leptomycin B (LMB), an antibiotic with anti-fungal and anti-tumor activity [32, 33]. LMB covalently modifies Cys528 in the cargo-binding groove of CRM1, which prevents the cargo-CRM1 interaction [20, 32]. In addition, the inhibitory effect of LMB on CRM1 is very selective as CRM1 is the only protein found to be conjugated by LMB in HeLa cells treated with LMB [32]. Importantly, LMB has been widely used to inactivate CRM1, leading to the identification of many CRM1 cargos [20, 35].

**Fig 2:**
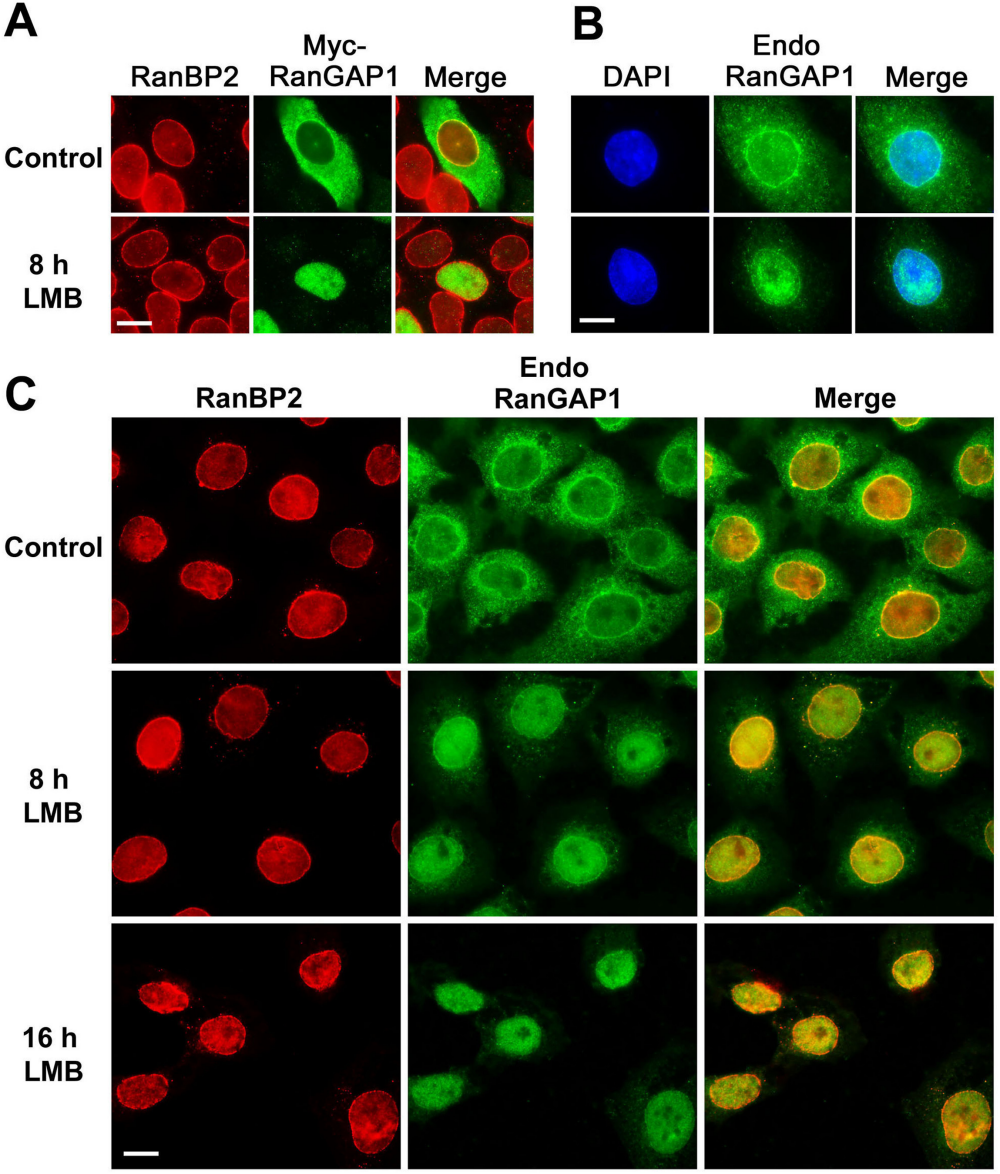
Inactivation of CRM1 by LMB causes nuclear accumulation of RanGAP1 and a loss or decrease of RanGAP1 distribution in the cytoplasm and at the NPC. (A) HeLa cells were transfected with the plasmids encoding Myc-tagged RanGAP1 for 24 h, treated with 20 nM LMB or a control solution (0.3% DMSO in PBS) for 8 h and analyzed by immunofluorescence microscopy using mouse anti-Myc mAb (9E10) and rabbit anti-RanBP2 antibodies. (B) BRL cells were treated with 20 nM LMB or a control solution for 8 h and analyzed by immunofluorescence microscopy with mouse anti-RanGAP1 mAb (19C7). (C) BRL cells were treated with either 20 nM LMB for 8 h and 16 h or a control solution for 16 h followed by immunofluorescence microscopy with antibodies specific to RanBP2 and RanGAP1. Bar, 10 μm.

To examine if inhibition of CRM1-mediated nuclear export by LMB treatment also caused nuclear accumulation of Myc-RanGAP1, HeLa cells transiently expressing Myc-RanGAP1 were treated with 20 nM LMB or a control solution for 8 h followed by immunofluorescence staining with antibodies specific to Myc and RanBP2 as a marker for the NPC. Compared to the predominant localization of Myc-RanGAP1 in the cytosol and at the NPC in control cells, LMB treatment resulted in a nuclear accumulation of Myc-RanGAP1 accompanied with a nearly complete loss of its distribution in the cytoplasm and at the NPCs (Fig 2A). To examine if LMB treatment has a similar effect on endogenous RanGAP1, BRL cells were treated with 20 nM LMB or a control solution for 8 h and then analyzed by immunofluorescence microscopy using anti-RanGAP1 mAb (19C7) [14] (Fig 2B). Compared to the primary localization of endogenous RanGAP1 in the cytoplasm and at the NPC in control cells, LMB treatment caused a nuclear accumulation of RanGAP1 accompanied with an obvious decrease of RanGAP1 in the cytoplasm and at the NPC (Fig 2B). Next, we tested if a prolonged treatment of BRL cells with LMB for 16 h instead of 8 h can cause a more robust redistribution of endogenous RanGAP1 from the NPC to the nucleoplasm in a way similar to Myc-RanGAP1 in response to 8 h of LMB treatment. To test this, BRL cells were treated with LMB for 8 h and 16 h or with a control solution followed by immunofluorescence microscopy (Fig 2C). Consistent with our hypothesis, we found that compared to 8 h of LMB treatment, 16 h of LMB treatment not only caused a predominantly nuclear accumulation of RanGAP1 but also nearly abolished its localization at the NPC and in the cytoplasm (Fig 2C). Hence, inhibition of CRM1-mediated export using CRM1 RNAi and LMB treatment caused a redistribution of both Myc-tagged and endogenous RanGAP1 from the cytoplasm and the NPC to the nucleoplasm, suggesting that CRM1 is responsible for the primary localization of RanGAP1 in the cytoplasm and at the NPC in mammalian cells.

### The NPC-associated RanGAP1 is more slowly redistributed to the nucleoplasm than the cytoplasmic RanGAP1 during LMB treatment

Our finding that Myc-RanGAP1 is almost completely redistributed from the cytoplasm and the NPC to the nucleoplasm after 8 h of LMB treatment prompted us to measure the reallocation rates of the cytoplasmic and NPC-associated Myc-RanGAP1 through a time-course analysis. While the cytoplasmic unmodified RanGAP1 is mobile, the NPC-associated SUMO1-modified RanGAP1 (RanGAP1*SUMO1) is immobilized in a stable RanBP2/RanGAP1*SUMO1/Ubc9 (RRSU) complex [12–15, 36]. The high stability of RanGAP1*SUMO1 at the NPC is supported by the finding that HA-tagged RanGAP1 remains stably associated with the NPC for 30 min in cells with plasma membrane permeabilized but maintaining an intact nuclear envelope during *in vitro* import assays, suggesting that RanGAP1*SUMO1 at the NPC is not deSUMOylated within this time frame [13]. On the other hand, incubation of HeLa cells with the bacterial virulence protein listeriolysin O (LLO) causes a specific degradation of the sole SUMO E2 enzyme Ubc9, dramatically reduces the half-life of Ubc9 from over 8 h to less than 10 min, and thus quickly blocks SUMOylation [37]. Upon 20 min of LLO treatment, the total amount of unmodified and SUMO1-modified RanGAP1 remains unchanged, but levels of unmodified RanGAP1 significantly increase, suggesting that SUMO1-modified RanGAP1 is deSUMOylated by SUMO-isopeptidases [37]. Furthermore, inhibition of SUMOylation by RNAi-knockdown of the sole SUMO E1 subunit SAE2 in U2OS cells markedly decrease in levels of SUMOylated RanGAP1 along with a corresponding increase in levels of unmodified RanGAP1 [38]. Moreover, a small fraction of SUMO1-modified RanGAP1 in complex with RanBP2 and Ubc9 is de-conjugated after a 25 min incubation with the SUMO isopeptidase Ulp1 *in vitro* [18]. However, it is still unclear about the dynamics of SUMO1-modified RanGAP1 at the NPC in cells.

**Fig 3:**
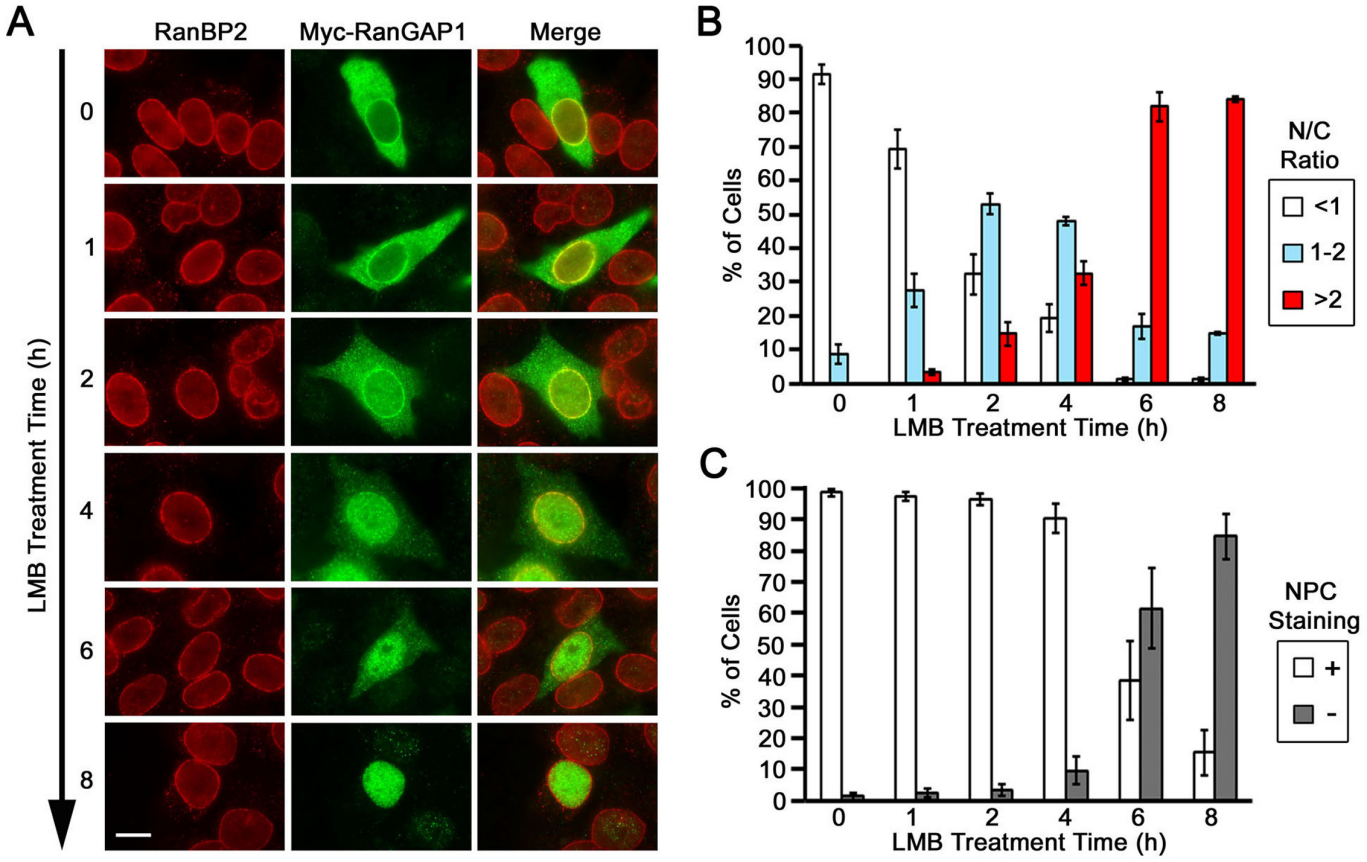
Time-course analysis of Myc-RanGAP1 redistribution during LMB treatment. (A) HeLa cells were transfected with the plasmids encoding Myc-tagged RanGAP1 for 24 h, treated with 20 nM LMB for the indicated times, and analyzed by immunofluorescence microscopy using anti-Myc (9E10) and anti-RanBP2 antibodies. Bar, 10 μm. (B) The histogram indicates the percentages of cells with the indicated N/C ratios of Myc-RanGAP1 during the time-course analysis from three independent experiments. Each bar represents the mean value ± SEM (*N* = 50, Student’s *t* test). (C) The histogram indicates the percentages of cells with (+) or without (-) NPC staining of Myc-RanGAP1 at each indicated time point (*N* = 50, Student’s *t* test). For each experiment, 50 cells at each time point were used to analyze N/C ratio and NPC staining of Myc-RanGAP1.

To address the above question, HeLa cells expressing Myc-RanGAP1 were treated with 20 nM LMB for 0, 1, 2, 4, 6 and 8 hours and analyzed by immunofluorescence microscopy with antibodies specific to RanBP2 and Myc (Fig 3A). We found that 2 h of LMB treatment not only decreased the percentage of Myc-RanGAP1 cells with an N/C ratio of <1 from ~92% (0 h) to ~32% (2 h) but also increased the percentage of cells with an N/C ratio of ≥1 from ~8% (1–2: 8%; >2: 0%) (0 h) to ~68% (1–2: 53%; >2: 15%) (2 h) (Fig 3B). This result suggests that inhibition of CRM1-mediated nuclear export over 2 h of LMB treatment causes ~60% of cells originally displaying a primarily cytoplasmic distribution of Myc-RanGAP1 to exhibit a nuclear accumulation of Myc-RanGAP1. Additionally, 6 h and 8 h of LMB treatment resulted in nearly all of the cells (~99%) displaying a nuclear accumulation of Myc-RanGAP1 with an N/C ratio of ≥1.

We further observed that 2 h and 4 h of LMB treatment only caused a small reduction in the percentage of Myc-RanGAP1 cells with an NPC staining from ~99% (0 h) to ~97% (2 h) and ~90% (4 h) (Fig 3C). However, 6 h and 8 h of LMB treatment resulted in a remarkable decrease in Myc-RanGAP1 cells with an NPC staining from 99% (0 h) to 38% (6 h) and to 15% (8 h) (Fig 3C). Based on our quantification results (Fig 3B and 3C), we estimated that ~1.5 h of LMB treatment might cause ~50% of cells with a primarily cytoplasmic distribution of Myc-RanGAP1 to display a nuclear accumulation of Myc-RanGAP1, and that ~5 h of LMB treatment might result in ~50% of cells to lose an NPC staining of Myc-RanGAP1. These findings suggested that the NPC-associated Myc-RanGAP1 is redistributed to the nucleoplasm much more slowly than the cytoplasmic Myc-RanGAP1 during LMB treatment. Compared to the cytoplasmic Myc-RanGAP1, the high stability of Myc-RanGAP1*SUMO1 in the RRSU complex at the NPC might be responsible for its much lower redistribution rate during LMB treatment. Furthermore, it is highly unlikely that the nearly complete loss or dramatic decrease of endogenous RanGAP1*SUMO1 and Myc-RanGAP1*SUMO1 at the NPC in LMB-treated cells (Figs 2 and 3) is caused by their degradation. It has been shown previously that there is no change in levels of RanGAP1*SUMO1 in HeLa cells after 4 h of cycloheximide treatment to block protein synthesis compared to control cells [39], suggesting that there is no obvious degradation of RanGAP1*SUMO1 at the NPC in a period of 4 h at least in HeLa cells.

### LMB treatment enforced RanGAP1 nuclear localization is reversible

**Fig 4:**
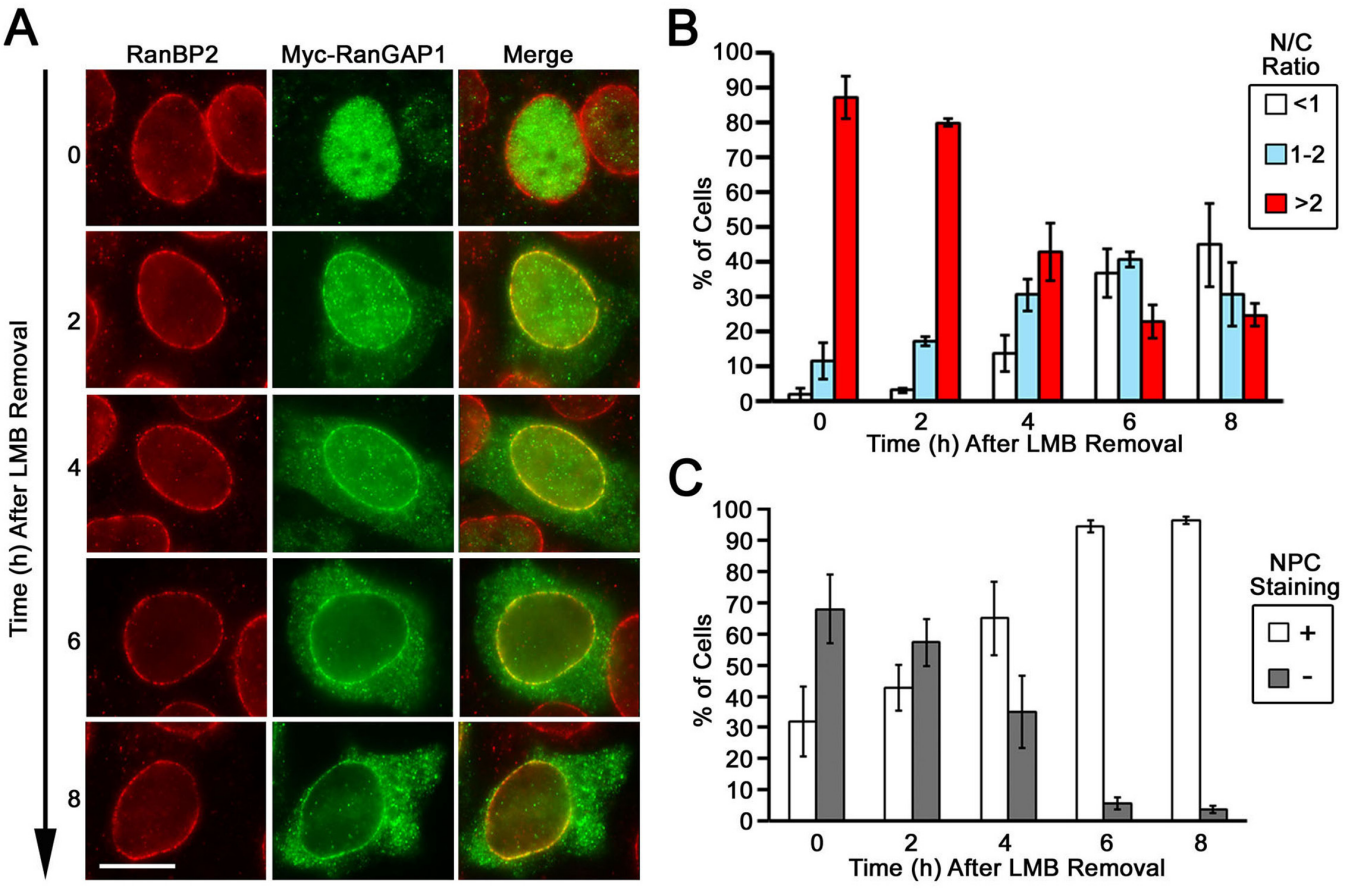
LMB-induced nuclear accumulation of Myc-RanGAP1 is reversible after removal of LMB. (A) HeLa cells transiently expressing Myc-tagged RanGAP1 were treated with 20 nM LMB for 8 h to induce a nuclear accumulation of RanGAP1. After removal of LMB, the cells were incubated with fresh medium for the indicated times and then analyzed by immunofluorescence microscopy using anti-RanBP2 and anti-Myc antibodies. Bar, 10 μm. (B) The histogram shows the percentages of cells with the indicated N/C ratios of Myc-RanGAP1 at each time point after removal of LMB. Each bar represents the mean value ± SEM (*N* = 50, Student’s *t* test). (C) The histogram indicates the percentages of cells with (+) or without (-) NPC staining of Myc-RanGAP1 at each time point after removal of LMB (*N* = 50, Student’s *t* test). The analyses were based on three independent experiments (B and C). For each experiment, 50 cells at each time point were used for analyzing both N/C ratio and NPC staining of Myc-RanGAP1.

To test if the nuclear accumulation of Myc-RanGAP1 in cells treated by LMB could be reversed, we incubated HeLa cells expressing Myc-RanGAP1 in LMB-free medium for 0, 2, 4, 6 and 8 h following LMB treatment for 6 h. In these experiments, we found that the predominantly nuclear Myc-RanGAP1 was gradually redistributed back to both the NPC and the cytoplasm after removal of LMB during a time period of 8 h (Fig 4A). The removal of LMB for 8 h increased the percentage of cells with a primarily cytoplasmic distribution of Myc-RanGAP1 from ~2% (0 h) to ~ 45% (8 h) as well as the percentage of cells with an NPC staining from ~32% (0 h) to ~ 96% (8 h) (Fig 4B and 4C). Hence, these findings revealed that LMB-induced nuclear accumulation of Myc-RanGAP1 is reversible after removal of LMB.

### LMB treatment increases SUMOylation of RanGAP1 in a RanBP2-independent fashion

Although vertebrate RanGAP1 is one of the best characterized SUMO substrates [12–16, 18, 19], it is still unclear where RanGAP1 is SUMOylated in cells. SUMOylation is catalyzed by an E1-activating enzyme (SAE1/SAE2), an E2-conjugating enzyme (Ubc9), and multiple E3 ligases [40–44]. All the essential components for SUMOylation, including SUMOs, SAE1/SAE2 and Ubc9, are predominantly nuclear, suggesting that SUMOylation mainly takes place in the nucleoplasm [30, 31, 45–48]. Consistent with this, addition of short sequences containing SUMOylation consensus motif (ΨKxE/D) (Ψ: a hydrophobic residue; K: the lysine residue for SUMOylation) to a non-SUMOylated carrier protein enables the *in vitro* SUMOylation of this fusion protein, but its SUMOylation *in vivo* requires the additional presence of an NLS for nuclear import [48]. Since our experiments revealed that vertebrate RanGAP1 shuttles between the cytoplasm and the nucleoplasm in a CRM1-dependent manner, we considered the possibility that RanGAP1 is imported into the nucleoplasm for efficient SUMOylation followed by the export and localization of SUMO-modified RanGAP1 at the cytoplasmic filaments of the NPC.

**Fig 5:**
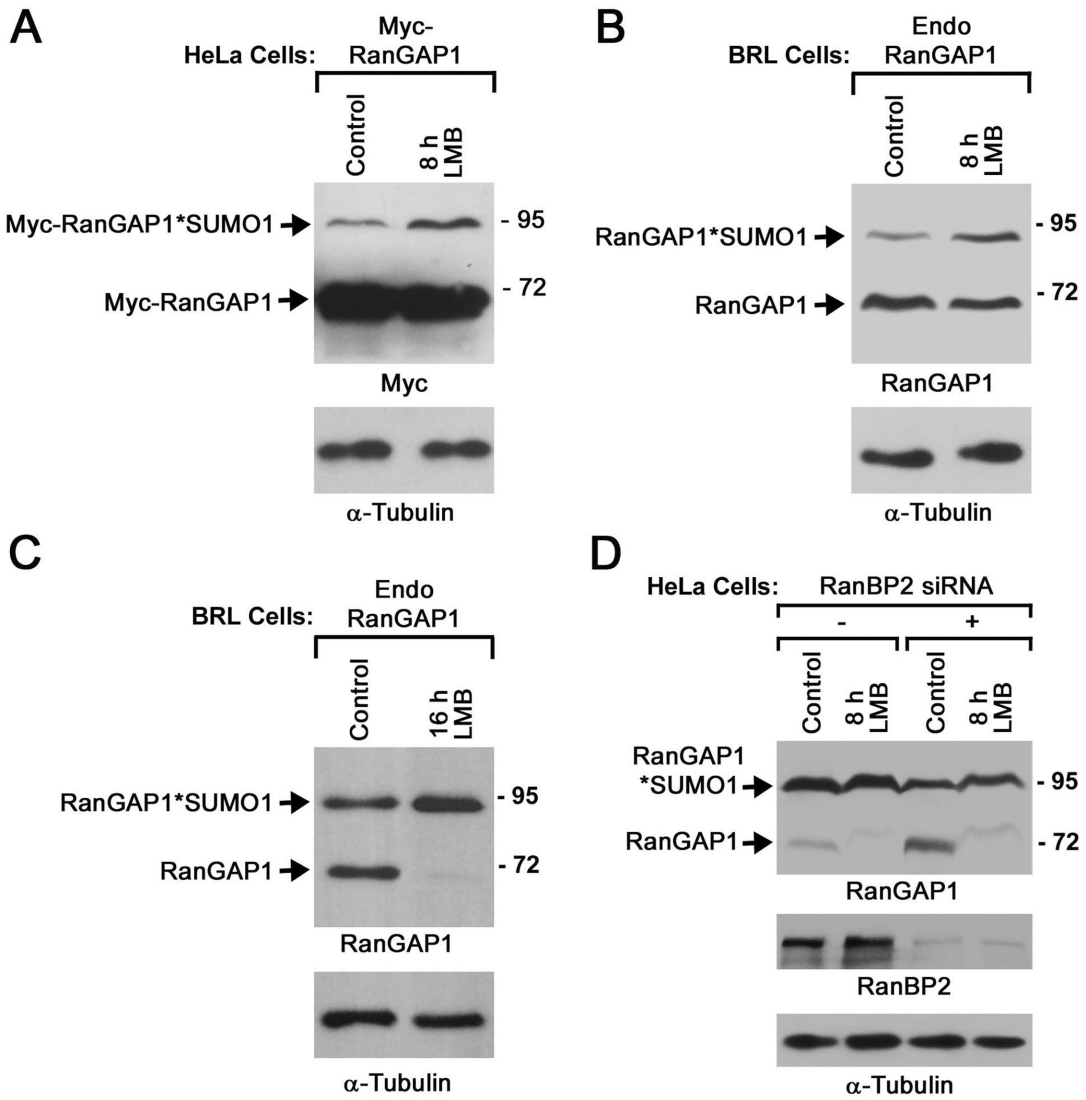
LMB treatment increases RanGAP1 SUMOylation in a RanBP2-independent manner. (A) HeLa cells were transfected with the plasmids encoding Myc-tagged RanGAP1 for 24 h, treated with 20 nM LMB or a control solution for 8 h, and analyzed by immunoblotting with mouse anti-Myc (9E10) and anti-α-tubulin mAbs. (B and C) BRL cells were treated with 20 nM LMB or a control solution for 8 h (B) and 16 h (C) followed by immunoblot analysis with mouse anti-RanGAP1 (19C7) and anti-α-tubulin mAbs. (D) HeLa cells were transfected with control or RanBP2-specific siRNA for 72 h, treated with 20 nM LMB or a control solution for 8 h, and then analyzed by immunoblotting with antibodies against RanGAP1, RanBP2 and α-tubulin.

Based on this model, we predicted that the nuclear accumulation of RanGAP1 caused by LMB treatment would also increase its SUMOylation. To test this, HeLa cells transiently expressing Myc-RanGAP1 were treated with 20 nM LMB or a control solution for 8 h and analyzed by immunoblotting with antibodies specific to Myc and α-tubulin. Compared to control treatment, LMB treatment markedly increased levels of SUMO-modified Myc-RanGAP1. At the same time, we did not observed an obvious decrease in levels of unmodified Myc-RanGAP1 in LMB-treated cells compared to control cells, which might be caused by saturated levels of unconjugated Myc-RanGAP1 detected in both cells (Fig 5A). To further test if LMB treatment also enhanced SUMOylation of endogenous RanGAP1, BRL cells were incubated with 20 nM LMB or a control solution for 8 h followed by immunoblot analysis with antibodies specific RanGAP1 and α-tubulin. We found that LMB treatment caused an obvious increase in levels of SUMO-modified RanGAP1 accompanied with a corresponding decrease in levels of unmodified RanGAP1 (Fig 5B). Next, we asked if a prolonged LMB treatment of BRL cells for 16 h other than 8 h can further enhance RanGAP1 SUMOylation. Consistent with this idea, we found that 16 h of LMB treatment caused an almost complete disappearance of unconjugated RanGAP1 along with a comparable increase of SUMO1-conjugated RanGAP1 compared to control cells (Fig 5C). Hence, we concluded that LMB treatment markedly increases SUMOylation of both endogenous and Myc-tagged RanGAP1.

RanBP2 forms a highly stable complex with SUMO1-modified RanGAP1 and Ubc9 at the NPC and therefore protects SUMO1-modified RanGAP1 from the SUMO-specific isopeptidase-mediated deSUMOylation [17, 18, 49]. Consistent with this model, RNAi-mediated depletion of RanBP2 in HeLa cells or conditional knockout of RanBP2 in mouse embryonic fibroblasts (MEF) greatly decreases levels of SUMO1-modified RanGAP1 along with a corresponding increase in levels of unmodified RanGAP1 [29, 50]. On the other hand, RanBP2 is a known SUMO E3 ligase for multiple proteins such as Sp100 but not RanGAP1 as indicated by *in vitro* SUMOylation assays using the internal repeat (IR) domain of RanBP2 [18, 51]. However, it is still unclear if the full length of endogenous RanBP2 acts as an E3 ligase for stimulating RanGAP1 SUMOylation *in vivo*. Our finding that LMB treatment increases RanGAP1 SUMOylation prompted us to ask if the increase in RanGAP1 SUMOylation in LMB-treated cells is dependent on RanBP2. To test this, we transfected HeLa cells with control siRNA or siRNA specific to RanBP2 for 72 h and then treated the transfected cells with 20 nM LMB or a control solution for 8 h followed by immunoblot analysis. In HeLa cells, a vast majority of RanGAP1 is present in its SUMO1-modified form, whereas only a very small fraction of RanGAP1 is found in its unmodified form [18, 29] (Fig 5D). This is in contrast to BRL cells, in which over 50% of RanGAP1 is present in its unmodified form [14] (Fig 5B and 5C). Consistent with previous studies [29, 50], RNAi-mediated knockdown of RanBP2 obviously increased levels of unmodified RanGAP1 (Fig 5D). In both control and RanBP2 RNAi cells, 8 h of LMB treatment caused an almost disappearance of unmodified RanGAP1 accompanied with an equivalent increase of SUMO1-modified RanGAP1 (Fig 5D). Hence, our results suggested that RanBP2 is not required for the LMB-induced increase of RanGAP1 SUMOylation in HeLa cells.

### The C-terminal domain of RanGAP1 contains a functional nuclear localization signal

**Fig 6:**
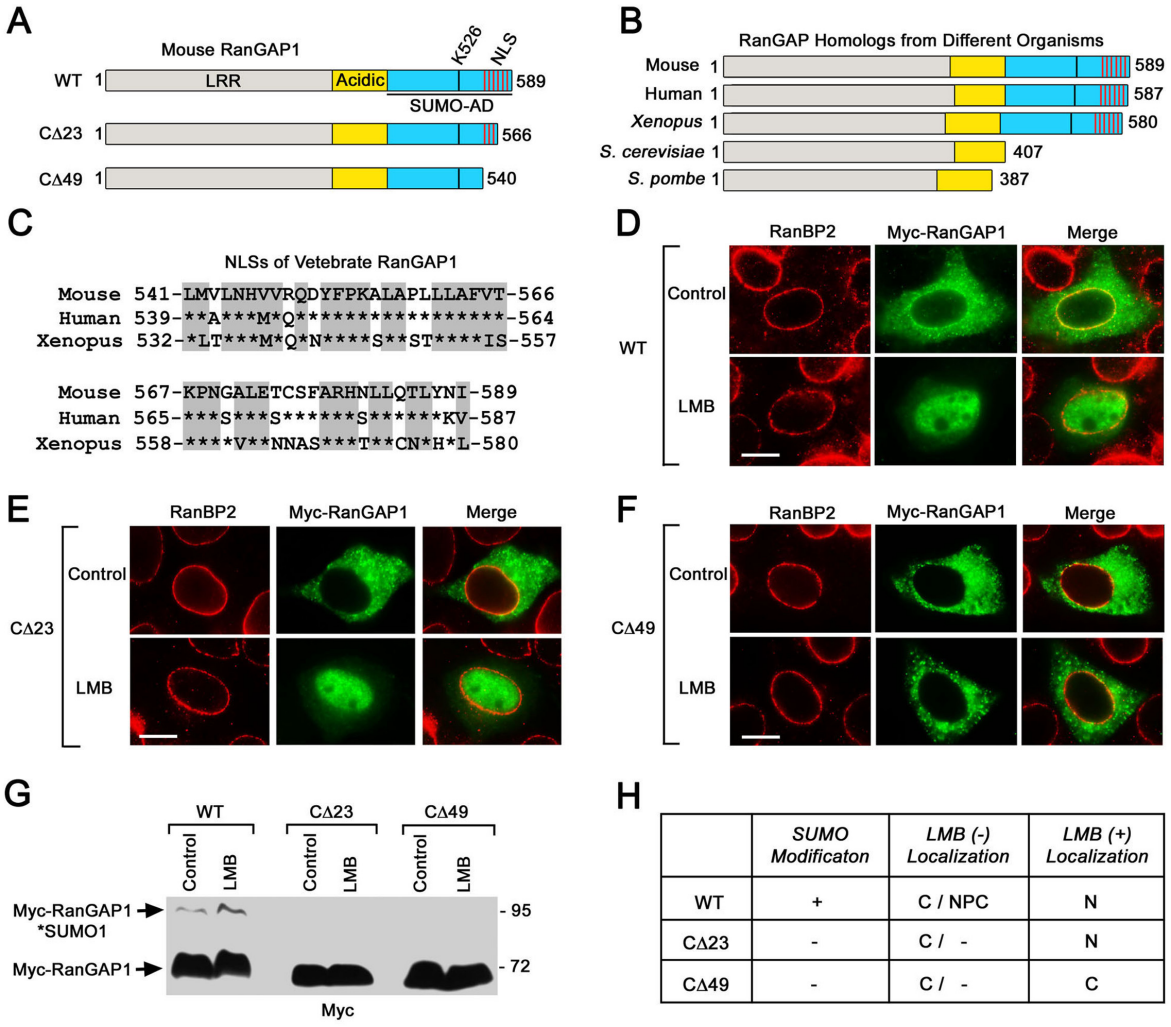
The putative NLS sequence at the C-terminus of RanGAP1 is required for its nuclear accumulation in cells treated with LMB. (A) The diagram shows mouse RanGAP1 wild-type (WT) (1–589) and its two C-terminal deletion mutants, CΔ23 (1–566) and CΔ49 (1–540). The mouse RanGAP1 consists of a leucine-rich repeat domain (LRR), an acidic region, and a SUMO-attachment domain (SUMO-AD) that contains a putative nuclear localization signal (NLS) (541–589) and also the lysine residue (K526) responsible for RanGAP1 SUMOylation [22–24]. (B) The alignment of RanGAP homologs, including mouse RanGAP1 (P46061.2), human RanGAP1 (P46060.1), *Xenopus* RanGAP1 (O13066.1), *S*. *cerevisiae* Rna1p (P11745.2) and *S*. *pombe* Rna1p (P41391.1). (C) The NLS sequence of mouse RanGAP1 (541–589) is aligned with those of human RanGAP1 (539–587) and *Xenopus* RanGAP1 (532–580). (D-G) HeLa cells were transfected with the constructs encoding Myc-tagged RanGAP1 WT (D), CΔ23 (E), and CΔ49 (F), treated with 20 nM LMB or a control solution for 8 h, and analyzed by immunofluorescence microscopy with antibodies against RanBP2 and Myc or by immunoblotting with anti-Myc antibody (G). (H) The chart summarizes the characteristics of RanGAP1 WT, CΔ23, and CΔ49 in SUMOylation and localization in response to LMB treatment. C: Cytoplasm; N: Nucleoplasm; NPC: nuclear pore complex. Bar, 10 μm.

Although our results show that the largely cytoplasmic distribution of RanGAP1 is maintained by CRM1-mediated nuclear export, it remains unclear how RanGAP1 enters the nucleus. Because the mammalian RanGAP1 is expressed as homodimer with a size of 150 kDa, which is too large for passive diffusion through the NPC, RanGAP1 is likely imported by importin(s). Consistent with this idea, Matunis and colleagues show that mouse RanGAP1 contains an NLS (541–589 amino acids) at its C-terminus (Fig 6A) as the fusion of this 541–589 sequence to pyruvate kinase (PK) targets the cytoplasmic PK to the nucleoplasm [12]. Interestingly, we found that the 541–589 sequence of mouse RanGAP1 shares ~69% similarities with those of human and *Xenopus* RanGAP1 (Fig 6B and 6C), suggesting that this NLS sequence might be conserved in vertebrates. However, it is still unclear if this NLS sequence of RanGAP1 is functional in regulating its own nuclear import.

To test this possibility, HeLa cells were transfected with the plasmids encoding Myc-tagged RanGAP1 wild-type (WT) (1–589) and its two C-terminal deletion mutants, CΔ23 (1–566) and CΔ49 (1–540), lacking the C-terminal 23 and 49 amino acids respectively (Fig 6A). The transfected cells were treated with 20 nM LMB or a control solution for 8 h followed by immunofluorescence microscopy and immunoblot analysis (Fig 6D–6G). In the absence of LMB treatment (control), both Myc-RanGAP1 CΔ23 and CΔ49 mutants displayed a primarily cytoplasmic distribution with no obvious staining at the NPC when compared to the predominant localization of Myc-RanGAP1 WT in the cytoplasm and at the NPC (Fig 6D–6H). The lack of NPC staining suggests that both CΔ23 and CΔ49 mutants are not SUMOylated *in vivo*. LMB treatment caused a nuclear accumulation of both Myc-RanGAP1 WT and CΔ23 mutant but not CΔ49 mutant (Fig 6D–6H). This result indicates that the C-terminal 49 amino acid sequence (541–589) of RanGAP1 is required for its own nuclear accumulation when CRM1-mediated nuclear export is inhibited by LMB treatment. Furthermore, our result also reveals that the 26 amino acid sequence (541–566), which is present in CΔ23 mutant (1–566) but deleted in CΔ49 mutant (1–540), likely represents a functional NLS as the CΔ23 mutant but not the CΔ49 mutant is accumulated in the nucleoplasm in cells treated with LMB.

To test if LMB treatment can enhance SUMOylation of Myc-RanGAP1 CΔ23 and CΔ49 mutants in a way similar to that of Myc-RanGAP1 WT, we analyzed SUMOylation of Myc-RanGAP1 WT, CΔ23 and CΔ49 in the presence or absence of LMB treatment. As expected, LMB treatment increased SUMOylation of WT, whereas SUMOylation of both mutants was not detected even in cells treated with LMB (Fig 6G). Consistent with our result, it has been shown previously that RanGAP1 CΔ23 mutant is not modified by SUMO *in vitro*, which is likely caused by its defect in interaction with the SUMO E2 enzyme Ubc9 [12, 52]. Because both CΔ23 and CΔ49 mutants are not SUMOylated in both control and LMB-treated cells (Fig 6G), we cannot use these two mutants to examine if RanGAP1 nuclear import affects its SUMOylation. On the other hand, the identification of the NLS sequence may allow us to generate the RanGAP1 mutant with a defect in nuclear import but not *in vitro* SUMOylation and Ubc9 interaction through site-directed mutagenesis. Such RanGAP1 mutant will enable us to further test if the nuclear import of RanGAP1 is critical for its SUMOylation.

## Discussion

By using both CRM1 RNAi and LMB treatment to inhibit CRM1-mediated nuclear export, we demonstrate that CRM1 is responsible for the primary distribution of RanGAP1 in the cytoplasm and at the NPC in mammalian cells. Furthermore, inhibition of CRM1-mediated export by LMB treatment not only causes a redistribution of RanGAP1 from the cytoplasm and the NPC to the nucleoplasm but also increases its SUMOylation. Intriguingly, it has been shown previously that SUMOylation of p53 promotes its nuclear export mediated by CRM1 [53]. Hence, it would be very interesting to investigate if SUMOylation of RanGAP1 also increases its CRM1-mediated nuclear export. Moreover, LMB treatment stimulates RanGAP1 SUMOylation in both control and RanBP2 RNAi cells, suggesting that the LMB-induced RanGAP1 SUMOylation is independent of the SUMO E3 ligase RanBP2. Lastly, we elucidate that the putative NLS sequence (541–589) at its C-terminus of RanGAP1 is required for its nuclear accumulation in LMB-treated cells and that the 26 amino acid sequence (541–566) of RanGAP1 may represent a functional NLS for its nuclear import. Altogether, our results support a model that SUMO1-modified RanGAP1 at the NPC is slowly deSUMOylated and released into the cytoplasmic pool of unmodified RanGAP1 and that the unmodified RanGAP1 is imported into the nucleus, SUMOylated in the nucleus, exported by CRM1 through the NPC, and targeted to the cytoplasmic filaments of the NPC by forming a complex with RanBP2 and Ubc9.

We notice that 8 h of LMB treatment causes ~99% of cells to display a nuclear accumulation of Myc-RanGAP1 (Fig 3B), whereas RNAi-knockdown of CRM1 using siRNA 1 or 2 results in only ~45% or ~30% of cells with a similar phenotype (Fig 1C). Furthermore, 8 h of LMB treatment also abolishes the localization of Myc-RanGAP1 at the NPC in ~85% of cells (Fig 3C), whereas CRM1 RNAi has no obvious effect on the NPC staining of Myc-RanGAP1 (Fig 1B). One explanation is that CRM1 RNAi only results in a partial knockdown of CRM1 compared to a nearly full inactivation of CRM1 by LMB. Despite its striking effect on Myc-RanGAP1 localization at the NPC (Figs 2A and 3A), 8 h of LMB treatment only decrease but does not abolish the localization of endogenous RanGAP1 at the NPC in BRL cells (Fig 2B). This might be due to the fact that only a very small fraction of Myc-RanGAP1 found in SUMOylated forms (Fig 5A), whereas a much higher percentage of endogenous RanGAP1 is SUMO1-modified and targeted to the NPC in BRL cells (Fig 5B and 5C) [12, 18]. Another possibility is that compared to endogenous RanGAP1*SUMO1, Myc-RanGAP1*SUMO1 may form a less stable complex with RanBP2 and Ubc9 at the NPC so that it is more easily dissociated and then de-conjugated followed by the nuclear import and localization of Myc-RanGAP1 in LMB-treated cells. Consistent with this, a prolonged LMB treatment of BRL cells for 16 h instead of 8 h increases the nuclear accumulation of endogenous RanGAP1 accompanied with a more robust decrease or nearly complete loss of endogenous RanGAP1 at the NPC and in the cytoplasm (Fig 2C).

Compared to control HeLa cells, the increase in levels of SUMO1-modified Myc-RanGAP1 in LMB-treated cells does not seem proportionally correlated to the nearly complete redistribution of Myc-RanGAP1 from the cytoplasm and the NPC to the nucleoplasm as most of Myc-RanGAP1 is still unmodified (Figs 2A and 5A). Consistent with previous studies [12, 18], only a small portion of Myc-RanGAP1 is modified by SUMO1 in HeLa cells, whereas over ~90% of endogenous RanGAP1 in HeLa cells is found in its SUMOylated form (Fig 5A and 5D). One possibility is that the addition of Myc tag to RanGAP1 may inhibit SUMOylation of RanGAP1 by altering the conformation of RanGAP1 and/or the interaction of RanGAP1 with Ubc9 and/or an unknown SUMO E3 ligase. The other possibility is that SUMO1-modifed Myc-RanGAP1 is more efficiently deSUMOylated by SUMO-isopeptidases compared to SUMO1-modified endogenous RanGAP1. The two possibilities are not mutually exclusive to each other. On the other hand, 16 h of LMB treatment causes a dramatic redistribution of endogenous RanGAP1 from the cytoplasm and the NPC to the nucleoplasm in BRL cells (Fig 2C). The predominant localization of RanGAP1 in the nucleoplasm in cells with 16 h of LMB treatment is accompanied with nearly all the RanGAP1 present in its SUMO-modified form when compared to control cells with only 50% of RanGAP1 found in its SUMOylated form (Figs 2C and 5C).

Our time-course analysis of LMB treatment shows that Myc-RanGAP1*SUMO1 is stably associated with the NPC but still slowly disassociates with a half-life of ~5 h at the NPC (Fig 3). Consistent with this, the RanGAP1*SUMO1 within the *in vitro* assembled RRSU complex is well protected from isopeptidase-mediated deSUMOylation, whereas a very small fraction of RanGAP1*SUMO1 is deSUMOylated after 25 min of incubation with isopeptidase [18]. There are two possibilities to explain the loss of Myc-RanGAP1*SUMO1 at the NPC during LMB treatment: one is that Myc-RanGAP1*SUMO1 transiently disassociates followed by its rapid deSUMOylation; the other is that it is gradually deSUMOylated within the complex and immediately disassociates. The stable association of Myc-RanGAP1*SUMO1 with the NPC might be responsible for its much slower redistribution rate to the nucleoplasm compared to the cytoplasmic Myc-RanGAP1 during LMB treatment.

In line with our finding that CRM1 is required for the primarily cytoplasmic localization of RanGAP1 in mammalian cells, inactivation of Crm1p in a *S*. *cerevisiae* strain harboring a temperature-sensitive mutation in *CRM1* gene by incubation of the cells at non-permissive temperature for 30 min causes nuclear accumulation of Rna1p [54]. The RanGAP proteins from various organisms, including *S*. *cerevisiae*, *S*. *pombe*, *Xenopus*, mouse and human, all contain leucine-rich NESs at their N-terminal LRR domains, suggesting that RanGAP is directly exported by CRM1 other than through an NES-containing adaptor protein [12, 54, 55]. The *S*. *pombe* Rna1p has been found to form the Rna1p-CRM1-RanGTP export complex in the presence of histone H3 *in vitro* [55]. The binding of histone H3 with Rna1p inhibits the RanGAP activity of Rna1p *in vitro*. In *S*. *pombe*, a segment of Rna1p is associated with chromatin and required for heterochromatin assembly by enhancing histone H3-lysine 9 (K9) methylation [55]. Therefore, it would be very interesting to examine if mammalian RanGAP1 play a similar role in the nucleoplasm.

Although the NLS sequence (541–589) of RanGAP1 can target the cytoplasmic pyruvate kinase to the nucleoplasm [12], little is known about its function in control of RanGAP1 localization. In this study, we show that the Myc-RanGAP1 CΔ49 mutant, which lacks the previously identified NLS sequence [12], is not accumulated in the nucleoplasm after 8 h of LMB treatment (Fig 6F). Our result indicates that this NLS sequence is required for the nuclear localization of RanGAP1 when CRM1-mediated nuclear export is inhibited by LMB treatment. Despite being uniquely localized at the C-terminal SUMO-AD domain of the vertebrate RanGAP1 (Fig 6B), the homologous NLS sequences are also found in RanGAP proteins from other organisms such as *S*. *cerevisiae* and *S*. *pombe*, which lack the C-terminal domain [54]. Hence, previous studies and ours suggest that both yeast Rna1 and mammalian RanGAP1 are actively imported into the nucleoplasm.

Given the essential role of RanGAP1 in nuclear transport by stimulating RanGTP hydrolysis in the cytoplasm, the nuclear accumulation of RanGAP1 caused by LMB treatment likely disrupts the RanGTP gradient and thus inhibit nuclear transport. Notably, studies of Segregation Distorter (*SD*), a meiotic drive system in *Drosophila* that preferentially transmit the *SD* chromosome from *SD/SD* ^+^ males to almost 100% of the progeny, have identified that the *Sd* locus encodes a truncated RanGAP (Sd-RanGAP) [56–58]. Although Sd-RanGAP has normal enzymatic activity, it is mislocalized to the nucleoplasm. In Sd-RanGAP cells, nuclear transport of a cargo reporter is disrupted, suggesting that the nuclear mislocalization of Sd-RanGAP perturbs nuclear transport by decreasing levels of RanGTP in the nucleus [57].

CRM1 is often overexpressed in tumor cells compared to the corresponding normal cells, and high CRM expression is also associated with a poor prognosis of cancer patients [59–63]. Upregulation of CRM1 in cancer cells results in the cytoplasmic accumulation of multiple tumor suppressor proteins, such as p53, p21, p27, APC, FOXO and BRCA1, and therefore disables their nuclear functions in preventing tumor initiation, growth and progression [64–69], suggesting that CRM1 is an attractive anti-cancer drug target. A family of CRM1 inhibitors, known as selective inhibitors of nuclear export (SINE), have been recently developed with strong anti-tumor activity and less toxicity compared to LMB [20, 70–72].

It has been hypothesized that cancer cells increase the rate of nuclear transport to keep up with their rapid growth and proliferation [67]. Consistent with this, four key nuclear transport factors, including CRM1, RanGAP1, Ran and RanBP1, are overexpressed in metastatic melanomas compared to primary melanomas and melanocytic nevi [63]. Further, RanGAP1 has been found as a potential marker and therapeutic target for aggressive B-cell lymphoma, especially diffuse large B-cell lymphoma (DLBCL) [73]. Because the primarily localization of RanGAP1 in the cytoplasm and at the NPC is required for efficient nuclear transport especially in cancer cells, targeting CRM1 with small-molecule inhibitors in certain types of cancers with overexpression of RanGAP1 and/or CRM1 might be a promising strategy for therapeutic treatment of these diseases.
